# Curcumin supplementation improves vascular endothelial function in healthy middle-aged and older adults by increasing nitric oxide bioavailability and reducing oxidative stress

**DOI:** 10.18632/aging.101149

**Published:** 2017-01-03

**Authors:** Jessica R. Santos-Parker, Talia R. Strahler, Candace J. Bassett, Nina Z. Bispham, Michel B. Chonchol, Douglas R. Seals

**Affiliations:** ^1^ Integrative Physiology, University of Colorado Boulder, Boulder, CO 80309, USA; ^2^ Renal Diseases and Hypertension, University of Colorado Denver, Aurora, CO 80045, USA

**Keywords:** aging, curcumin, endothelium-dependent dilation, arterial stiffness, oxidative stress, inflammation

## Abstract

We hypothesized that curcumin would improve resistance and conduit artery endothelial function and large elastic artery stiffness in healthy middle-aged and older adults. Thirty-nine healthy men and postmenopausal women (45-74 yrs) were randomized to 12 weeks of curcumin (2000 mg/day Longvida®; n=20) or placebo (n=19) supplementation. Forearm blood flow response to acetylcholine infusions (FBF_ACh_; resistance artery endothelial function) increased 37% following curcumin supplementation (107±13 vs. 84±11 AUC at baseline, P=0.03), but not placebo (P=0.2). Curcumin treatment augmented the acute reduction in FBF_ACh_ induced by the nitric oxide synthase inhibitor *NG* monomethyl-L-arginine (L-NMMA; P=0.03), and reduced the acute increase in FBF_ACh_ to the antioxidant vitamin C (P=0.02), whereas placebo had no effect (both P>0.6). Similarly, brachial artery flow-mediated dilation (conduit artery endothelial function) increased 36% in the curcumin group (5.7±0.4 vs. 4.4±0.4% at baseline, P=0.001), with no change in placebo (P=0.1). Neither curcumin nor placebo influenced large elastic artery stiffness (aortic pulse wave velocity or carotid artery compliance) or circulating biomarkers of oxidative stress and inflammation (all P>0.1). In healthy middle-aged and older adults, 12 weeks of curcumin supplementation improves resistance artery endothelial function by increasing vascular nitric oxide bioavailability and reducing oxidative stress, while also improving conduit artery endothelial function.

## INTRODUCTION

Cardiovascular diseases (CVD) are the primary cause of death in developed societies [1, 2]. Advancing age is the major risk factor for CVD, with a ∼70% prevalence of CVD in men and women over 60 years of age [1, 3]. This increase in CVD risk with aging is due primarily to adverse changes to arteries, in particular, the development of vascular endothelial dysfunction and increased stiffness of large elastic arteries [4].

Both resistance artery (microvascular) and conduit artery (macrovascular) endothelial function, as measured by endothelium-dependent dilation (EDD), decline with advancing age [5–11] and each is independently predictive of future risk of cardiovascular events and mortality [12–15]. Vascular endothelial dysfunction with age appears to develop first in resistance vessels and subsequently in the conduit arteries [5, 9, 16]. A key mechanism mediating the development of age-related endothelial dysfunction is reduced bioavailability of the vascular protective and vasodilatory molecule, nitric oxide [10, 17–19]. Decreased nitric oxide bioavailability with age is in part driven by the presence of oxidative stress, an increase in reactive oxygen species relative to antioxidant defenses, and chronic low-grade inflammation [8, 20–22].

Stiffness of large elastic arteries (aorta and carotid arteries) is commonly assessed regionally and locally by aortic pulse wave velocity (PWV) and carotid artery compliance, respectively [23, 24]. Large elastic arteries stiffen with advancing age [25–28], which is associated with a higher risk of cardiovascular-related mortality [29–31]. Changes in vascular smooth muscle tone and structural components of the arterial wall are thought to be the predominate factors contributing to the increase in stiffness [4, 32, 33], driven by reductions in nitric oxide bioavailability and increases in oxidative stress and inflammation [34–36]. With the number of older adults in the United States expected to double by the year 2050 [37], interventions that improve age-related vascular endothelial dysfunction and arterial stiffness are needed to reduce the risk of CVD in this growing population.

Curcumin is a naturally occurring phenol found in the Indian spice turmeric. Curcumin has been reported to increase nitric oxide production and reduce oxidative stress and inflammation in cell and animal models of vascular-related disease [38–46], as well as healthy and diseased human populations [47–50]. In a recent preclinical study from our laboratory [51], we demonstrated that 4 weeks of curcumin supplementation improved conduit artery endothelial function in older male mice to levels of young animals, mediated by an increase in nitric oxide bioavailability and a reduction in vascular oxidative stress. In addition, curcumin supplementation ameliorated aortic stiffening, as indicated by reductions in aortic PWV to that of young

adult mice. Taken together, these data suggest that curcumin supplementation holds promise as a treatment strategy for age-related arterial dysfunction.

The purpose of this study was to translate our preclinical findings in older mice to healthy middle-aged and older adults. We hypothesized that curcumin supplementation would improve age-related vascular endothelial function in middle-aged and older men and postmenopausal women by increasing nitric oxide bioavailability secondary to a reduction in oxidative stress, while also improving large elastic artery stiffness and, possibly, markers of systemic inflammation. To test this hypothesis, we performed a double-blind, parallel design, randomized study in which thirty-nine participants received curcumin (2000 mg/day Longvida® pill) or placebo supplementation for 12 weeks. Resistance artery EDD in the absence and presence of intact nitric oxide production and oxidative stress was assessed, along with conduit artery EDD, large elastic artery stiffness, and circulating markers of oxidative stress and inflammation.

## RESULTS

### Participants

**Figure 1:**
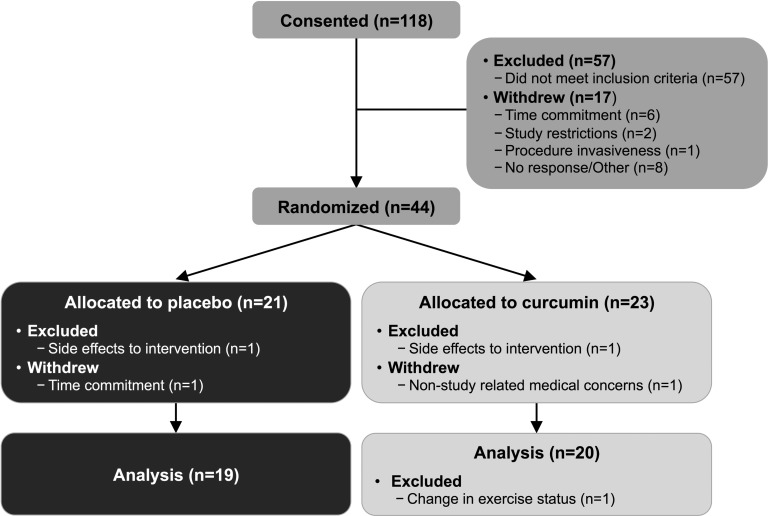
Participant progress through study.

One hundred and eighteen participants were consented for the study. Fifty-seven individuals did not meet inclusion criteria. Seventeen individuals withdrew from the study prior to randomization due to the time commitment (n=6), study restrictions (n=2), procedure invasiveness (n=1), or did not respond to scheduling requests (n=8). Twenty-one participants were randomized to the placebo group and twenty-three participants to the curcumin group. Two placebo group participants did not complete the study (excluded n=1, side effects: gastrointestinal discomfort; withdrew n=1, time commitment). Two curcumin group participants did not complete the study (excluded n=1, side effects: dizziness; withdrew n=1, non-study related medical concerns) and one participant was excluded from analysis due to a change in exercise status (Figure 1). Completed participants were of non-Hispanic Caucasian (n=32), non-Hispanic Asian (n=3), Hispanic Caucasian (n=2), non-Hispanic African American (n=1), or non-Hispanic American Indian/Alaskan (n=1) ethnicity.

### Participant characteristics

All participant characteristics (sex, age, body mass index, waist to hip ratio, body fat percent, blood pressure, heart rate, maximal oxygen consumption, physical activity energy expenditure, lipids, glucose, C-reactive protein) were not different between the placebo and curcumin groups at baseline (all P>0.3), except for body mass, which was higher in the placebo group (P=0.03). Participants were sedentary or moderately physically active. No participant characteristics changed with time between groups (all P>0.05; Table 1).

**Table 1:** Participant characteristics

	Placebo		Curcumin	
	Week 0	Week 12	Week 0	Week 12
N, men/women	11/8	–	10/10	–
Age, years	61±2	–	63±2	–
Body mass, kg	76±3*	75±3	68±2	68±3
Body mass index, kg/m^2^	25±1	25±1	24±1	24±1
Waist to hip ratio, U^L^	0.81±0.05	0.85±0.02	0.84±0.02	0.84±0.02
Body fat, %	27.9±2.0	27.8±2.0	30.1±1.9	30.2±1.9
Systolic blood pressure, mmHg	120±3	122±3	121±3	121±3
Diastolic blood pressure, mmHg	73±2	73±1	72±1	71±1
Resting heart rate, beats/min^L^	56±2	55±2	55±1	57±2
VO_2_ max, mL/kg/min	33±1	33±1	31±1	31±1
Physical activity energy expenditure, kcal/wk^L^	6095±872	5334±819	5720±954	5416±984
Total cholesterol, mg/dL	177±6	173±6	175±8	174±6
HDL-cholesterol, mg/dL^L^	56±4	52±4	55±5	56±4
LDL-cholesterol, mg/dL	103±6	102±6	103±7	101±6
Triglycerides, mg/dL^L^	97±15	93±15	86±11	91±12
Glucose, mg/dL	84±2	85±2	85±2	87±2
C-reactive protein, mg/L^L^	0.96±0.26	1.15±0.34	0.81±0.14	0.72±0.12

Data are mean±SE; HDL, high-density lipoprotein; LDL, low-density lipoprotein;

L Data log transformed for statistical analysis;

* P=0.03 vs. curcumin week 0

### Circulating humoral factors

Circulating humoral factors were assessed in a subset of participants (placebo n=11-12, curcumin n=13-14) and are presented in Supplemental Table 1. All circulating blood factors (interleukin-6: IL-6, tumor necrosis factor-α: TNF-α, oxidized low-density lipoprotein: oxidized LDL, total antioxidant status: TAS, glutathione peroxidase, epinephrine, norepinephrine, endothelin-1, cortisol, adiponectin, leptin, insulin, and homeostasis model assessment of insulin resistance: HOMA-IR) were not different between the placebo and curcumin groups at baseline (all P>0.1), except for free fatty acids, which were lower in the placebo group (P=0.03). No circulating humoral factors changed with time between groups (all P>0.1).

### Curcumin safety and tolerability

72% of participants did not miss any intervention pills. Of the 11 participants who missed pills, 5 participants were in the curcumin group and missed a total of 4 to 12 pills throughout the intervention. No severe or unexpected adverse events occurred and the 2000 mg/day Longvida® formulation was well tolerated. Three curcumin group participants experienced “expected” adverse events, including diarrhea (n=1), dizziness (n=1), and gastrointestinal discomfort (n=1). The participant experiencing dizziness was excluded from the study. Four placebo group participants also experienced adverse events, including gastrointestinal discomfort (n=2), diarrhea (n=1), and nausea (n=1). The placebo group participant who experienced gastrointestinal discomfort was excluded from the study (Table 2).

**Table 2:** Safety and tolerability

	Placebo	Curcumin
Treatment-related adverse events, N		
Diarrhea	1	1
Dizziness	0	1
Gastrointestinal discomfort	2	1
Nausea	1	0
Subjects with ≥ 1 adverse event, n	0	0
Excluded, N	1	1

### Dietary analysis

Total daily energy, relative carbohydrate, and relative fat intake were not different between the placebo and curcumin groups at baseline (all P>0.3) except for daily relative protein intake, which was slightly higher in the placebo group (P=0.02). No dietary intake factors changed with time between the placebo and curcumin groups (all P>0.05; Supplemental Table 2).

### Resistance artery endothelial function

**Figure 2:**
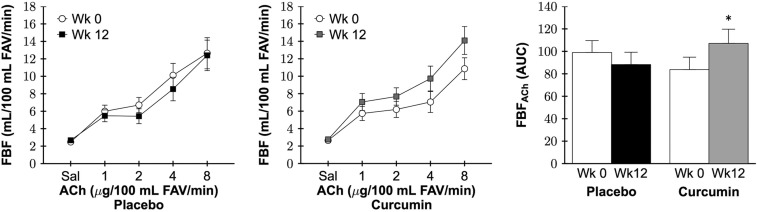
Forearm blood flow (FBF) in response to increasing doses (*left and middle*) and area under the dose-response curve (AUC; *right*) to acetylcholine (FBF_ACh_) at week 0 and after 12 weeks of placebo or curcumin supplementation. Data are mean ± SE; FAV, forearm volume; Group by time P=0.02, *P=0.03 vs. curcumin week 0.

**Figure 3:**
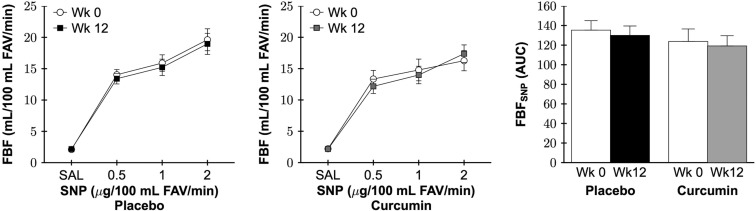
Forearm blood flow (FBF) in response to increasing doses (*left and middle*) and area under the dose-response curve (AUC; *right*) to sodium nitroprusside (FBF_SNP_) at week 0 and after 12 weeks of placebo or curcumin supplementation. Data are mean±SE; FAV, forearm volume; Group by time P=0.9.

Forearm blood flow response to incremental intrabrachial artery infusion of acetylcholine (FBF_ACh_) was assessed as a measure of resistance artery endothelial function in a subset of participants (n=12 per group) due to difficulty placing intra-arterial lines in all participants both before and after the intervention period. FBF_ACh_ area under the dose-response curve (AUC) was not different between the placebo and curcumin groups at baseline (P=0.3). FBF_ACh_ AUC had a group by time interaction between the placebo and curcumin supplementation groups (P=0.02). FBF_ACh_ AUC increased 37% after 12 weeks of curcumin supplementation (P=0.03), whereas there was no change with placebo (P=0.2; Figure 2). Individual FBF_ACh_ AUC at baseline and week 12 for each group are presented in Supplemental Figure 1. FBF_ACh_ AUC was higher after 12 weeks of curcumin vs. baseline in 9 of the 12 participants treated with curcumin compared with only 2 of the 12 placebo group participants. No sex differences in FBF_ACh_ AUC in the curcumin-supplemented group were observed (P=0.2). Resistance artery endothelium-independent dilation, a measure of vascular smooth muscle sensitivity to nitric oxide assessed as the increase in FBF in response to the nitric oxide donor sodium nitroprusside (FBF_SNP_), was not different between the placebo and curcumin groups at baseline (P=0.5) and did not change with time between groups (P=0.9; Figure 3).

### Resistance artery nitric oxide-mediated endothelial function

**Figure 4:**
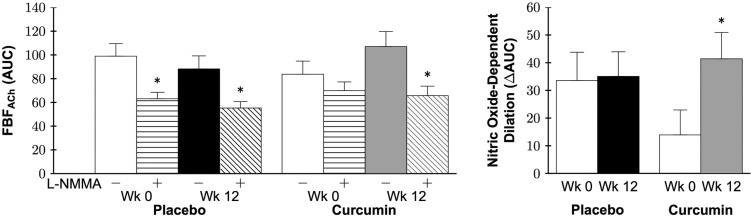
Forearm blood flow (FBF) area under the dose-response curve (AUC) in response to acetylcholine (FBF_ACh_) without (−) or with (+) *NG* monomethyl-L-arginine (L-NMMA; *left*) and nitric oxide-dependent dilation (*right*) at week 0 and after 12 weeks of placebo or curcumin supplementation. Data are mean±SE; *P<0.02 vs. corresponding group week FBF_ACh_ (*left*); Group by time P=0.04, *P=0.03 vs. curcumin week 0 *(right*).

As shown in Figure 4 left, in the placebo group there were similar reductions in FBF_ACh_ AUC with co-infusion of the nitric oxide synthase inhibitor NG monomethyl-L-arginine (L-NMMA) at week 0 and week 12 (both P=0.002). In contrast, in the curcumin-treated group, reduction in FBF_ACh_ with L-NMMA at week 12 following curcumin supplementation (P=0.001) was more significant than that observed at week 0 (P=0.08). To illustrate the effect of curcumin supplementation on the contribution of nitric oxide to improvements in FBF_ACh_, nitric oxide-dependent dilation was calculated as FBF_ACh_ with L-NMMA - FBF_ACh_ (ΔAUC) and presented as positive values in Figure 4 right. There was a distinct group by time interaction between the placebo and curcumin supplementation groups (P=0.04). Specifically, 12 weeks of curcumin supplementation increased nitric-oxide dependent dilation (P=0.03), whereas no change was observed in the placebo group (P=0.7).

### Resistance artery oxidative stress-mediated suppression of endothelial function

**Figure 5:**
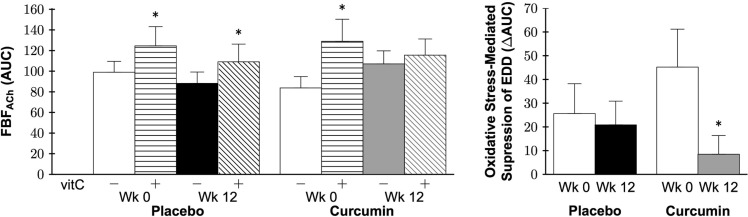
Forearm blood flow (FBF) area under the dose-response curve (AUC) in response to acetylcholine (FBF_ACh_) without (−) or with (+) vitamin C (vitC; *left*) and oxidative stress-mediated suppression of endothelium-dependent dilation (EDD; *right*) at week 0 and after 12 weeks of placebo or curcumin supplementation. Data are mean±SE; *P<0.05 vs. corresponding group week FBF_ACh_ (*left*); Group by time P=0.03, *P=0.02 vs. curcumin week 0 *(right*).

Co-infusion of the antioxidant vitamin C increased FBF_ACh_ in both groups at baseline (both P<0.05), demonstrating a tonic oxidative stress-mediated suppression of resistance artery endothelial function. At 12 weeks, co-infusion of vitamin C did not improve FBF_ACh_ in the curcumin-supplemented group (P=0.3), but did improve FBF_ACh_ in the placebo group (P=0.03; Figure 5 left). To illustrate the influence of curcumin supplementation, oxidative stress-mediated suppression of EDD was calculated as FBF_ACh_ with vitamin C − FBF_ACh_ (ΔAUC) and presented in Figure 5 right. A group by time interaction between the placebo and curcumin supplementation groups was observed (P=0.03). Specifically, 12 weeks of curcumin supplementation reduced oxidative stress suppression of EDD (P=0.02), whereas no change was observed in the placebo group (P=0.6).

### Conduit artery endothelial function

**Figure 6:**
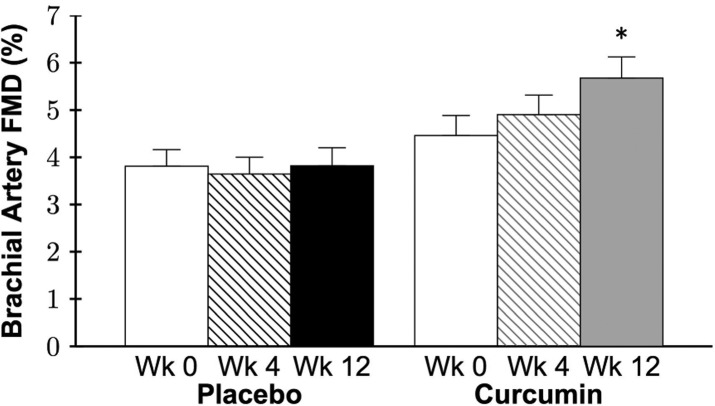
Brachial artery flow-mediated dilation (FMD) expressed as percent change at week 0 and after 4 and 12 weeks of placebo or curcumin supplementation. Data are mean±SE; Group by time P=0.001, *P=0.001 vs. curcumin week 0.

**Figure 7:**
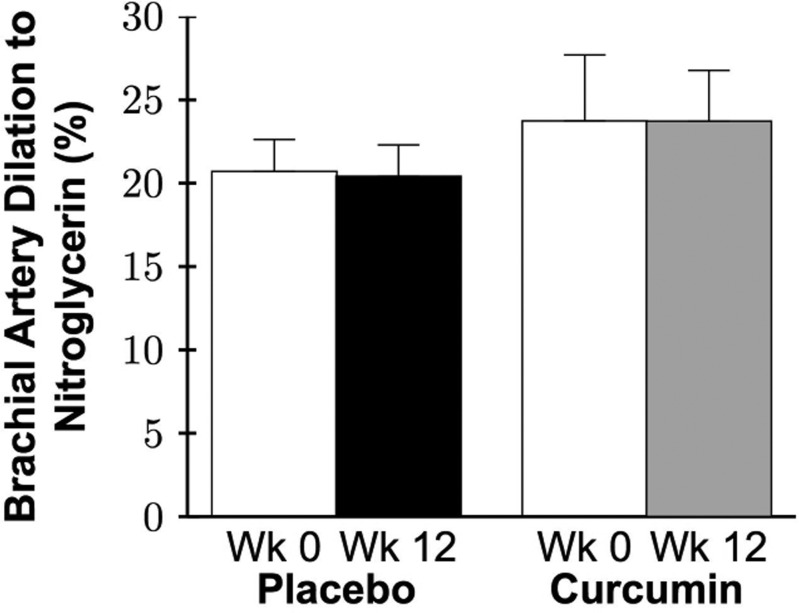
Brachial artery dilation to nitroglycerin expressed as percent change at week 0 and after 12 weeks of placebo or curcumin supplementation. Data are mean±SE; Group by time P=0.8.

No group differences in brachial artery flow-mediated dilation (FMD; Figure 6), a measure of conduit artery EDD, or parameters (Table 3) were observed at baseline (all P>0.05). A group by time interaction was observed in brachial artery FMD percent (P=0.001) and absolute (P=0.001) change in which 12 weeks of curcumin supplementation increased brachial artery FMD 36% (P=0.001) with no changes in the placebo group (P=0.8; Figure 6; Table 3). Individual brachial artery FMD at baseline, week 4, and week 12 for each group is presented in Supplemental Figure 2. Brachial artery FMD was higher after 12 weeks of curcumin vs. base-line in 17 of the 20 subjects treated with curcumin compared with only 8 of the 19 placebo group subjects. A strong trend for an improvement in FMD was observed at 4 weeks in the curcumin group (P=0.09), but not in the placebo group (P=0.2). Brachial artery dilation to nitroglycerin, a measure of conduit artery endothelium-independent dilation, was assessed in a subset of participants (placebo n=9, curcumin n=6) due to safety restrictions in administering nitroglycerin to individuals with low blood pressure or history of migraines. There were no significant effects of treatment on brachial artery dilation to nitroglycerin (P=0.8; Figure 7). In the curcumin-supplemented group, a sex by time interaction in brachial artery FMD was observed at 12 weeks (P=0.001), with significant improvements in both sexes (week 12 vs. baseline: men P=0.001, women P=0.01), but a greater magnitude of improvement in men compared with women (Supplemental Figure 3).

**Table 3:** Brachial artery parameters

	Placebo			Curcumin		
	Week 0	Week 4	Week 12	Week 0	Week 4	Week 12
Baseline diameter, mm	3.77±0.18	3.76±0.18	3.76±0.17	3.40±0.14	3.42±0.15	3.38±0.15
FMD absolute change, mm	0.14±0.01	0.13±0.01	0.14±0.01	0.14±0.01	0.16±0.01	0.19±0.02*
Peak diameter, mm	3.90±0.18	3.89±0.18	3.90±0.17	3.54±0.13	3.58±0.15	3.56±0.15
Time to peak diameter, s^L^	38±2	38±3	37±3	40±3	41±4	40±3
FMD shear rate, s^−1^^L^	1885±149	1816±190	1908±183	1977±166	2056±177	2150±170
Nitroglycerin dilation, mm^#^	0.79±0.05	–	0.77±0.04	0.77±0.10	–	0.78±0.08

Data are mean±SE; FMD, flow-mediated dilation;

# Placebo, n=9, curcumin, n=6;

L Data log transformed for statistical analysis; Group by time P=0.001

* P=0.001 vs. curcumin week 0

### Arterial stiffness

**Figure 8:**
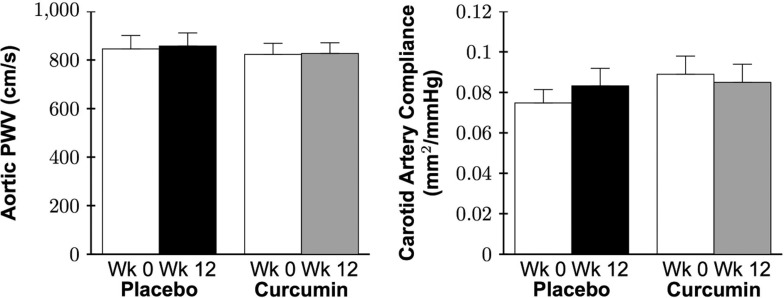
Aortic pulse wave velocity (PWV; *left*) and carotid artery compliance (*right*) at week 0 and after 12 weeks of placebo or curcumin supplementation. Data are mean±SE; Data log transformed for statistical analysis; Both group by time P>0.2.

There were no baseline group differences in aortic PWV, carotid artery compliance (Figure 8), or other arterial stiffness parameters (all P>0.1; Table 4), except for change in carotid artery diameter, which was lower in the placebo group (P=0.02). There was no group by time interaction for aortic PWV (P=0.8), carotid artery compliance (P=0.2), or other arterial stiffness parameters (all P>0.2). No sex-differences in aortic PWV or carotid artery compliance in the curcumin-supplemented group were observed (both P>0.3).

**Table 4:** Arterial stiffness parameters

	Placebo		Curcumin	
	Week 0	Week 12	Week 0	Week 12
Carotid systolic blood pressure, mmHg	117±5	116±5	123±4	123±4
Carotid pulse pressure, mmHg	49±3	48±3	56±4	56±3
Carotid artery diameter at end-diastole, mm^L^	6.35±0.2	6.35±0.2	6.41±0.1	6.38±0.2
Carotid change in diameter, mm	0.35±0.02*	0.36±0.02	0.45±0.03	0.44±0.03
Carotid augmentation index, %	16±3	16±3	17±2	17±2
Carotid β-stiffness index, U	10.3±0.7	9.4±0.5	9.1±0.6	9.2±0.5
Carotid to radial PWV, cm/s	991±26	989±34	945±34	930±47
Radial augmentation index, %	−13±3	−12±3	−8±3	−6±3
Carotid IMT at end diastole, mm^L^	0.56±0.01	0.56±0.01	0.60±0.02	0.60±0.02

Data are mean±SE; PWV, pulse wave velocity; IMT, intima-media thickness;

L Data log transformed for statistical analysis;

* P=0.02 vs. curcumin week 0

## DISCUSSION

This is the first study in humans to assess the beneficial effects of curcumin supplementation on resistance and conduit artery endothelial function, regional and local large elastic artery stiffness, and the mechanisms involved. The present findings demonstrate that 12 weeks of curcumin supplementation is safe and well tolerated, and improves resistance and conduit artery endothelial function in healthy middle-aged and older adults. Improvements in both vascular beds were endothelium specific, as no changes in endothelium-independent dilation were observed. As assessed in resistance vessels, the improvements in endothelial function were mediated by an increase in nitric oxide bioavailability and a reduction in vascular oxidative stress. No changes in regional or local large elastic artery stiffness or circulating biomarkers of oxidative stress and inflammation were observed after 12 weeks of curcumin supplementation.

### Curcumin supplementation and vascular endothelial function

Resistance and conduit artery nitric oxide-mediated endothelial function declines with advancing age [5–11], with resistance artery dysfunction believed to precede conduit artery impairments [5, 9, 16]. Measures of resistance and conduit artery endothelial function, FBF_ACh_ and brachial artery FMD, respectively, are each independently predictive of future risk of a cardio-vascular event or mortality [12–15], although not necessarily correlated to one another [52]. When considered along with differences between conduit and resistance vessel structure and function [53], this suggests that each vascular bed may have relevance to different aspects of CVD and emphasizes the importance of assessing both the resistance and conduit vasculature health.

#### Resistance artery function, nitric oxide bioavailability, and oxidative stress

To our knowledge, no studies have assessed the effects of curcumin supplementation on resistance artery endothelial function in the context of primary aging in preclinical models or humans. In rodent models of diabetes and hypertension, beneficial effects of curcumin on resistance artery endothelial function have been reported in the heart, brain, and eye [54–56]. In the current study, we show that 12 weeks of oral curcumin supplementation improves resistance artery endothelial function in healthy middle-aged and older men and postmenopausal women. These improvements in FBF_ACh_ were endothelium specific, as no changes in FBF_SNP_ were observed. 12 weeks of curcumin supplementation enhanced the acute reduction in FBF_ACh_ induced by co-infusion with the nitric oxide synthase inhibitor L-NMMA, indicating that improve-ments in resistance artery endothelial function were mediated by an increase in nitric oxide bioavailability.

In our recent preclinical study [51], improvements in *ex vivo* carotid artery EDD with 12 weeks of curcumin supplementation in older mice were associated with reduced oxidative stress, as acute *ex vivo* administration of the superoxide dismutase mimetic, TEMPOL, restored EDD in older non-supplemented mice but had no effect on EDD in older curcumin-supplemented mice. Consistent with these observations, in the present study 12 weeks of curcumin supplementation reduced oxidative-stress mediated suppression of resistance artery endothelial function, as evidenced by the smaller improvement in the FBF_ACh_ in response to co-infusion of the antioxidant vitamin C following curcumin supplementation. Taken together, these observations indicate that a reduction in vascular oxidative stress was a key mechanism underlying improvements in nitric oxide-mediated resistance artery endothelial function after 12 weeks of curcumin supplementation in our healthy middle-aged and older participants.

#### Conduit artery function

Curcumin is reported to protect against or improve conduit artery endothelial dysfunction in animal models of cardio-metabolic disease, including diabetes, hypertension, and metabolic syndrome [45, 46, 57, 58]. However, research assessing the effects of curcumin on age-related conduit artery endothelial dysfunction has been limited.

A preclinical study performed by our laboratory [51] demonstrated that 4 weeks of curcumin-supplemented chow restored nitric oxide-mediated *ex vivo* carotid EDD in older (26-28 months) male mice to levels of young (4-6 months), with no effect in young mice. In humans, 8 weeks of curcumin supplementation improved brachial artery FMD in healthy Japanese postmenopausal women [59], similar to a new report in young adults [60].

In agreement with these findings, we found that 12 weeks of curcumin supplementation improved brachial artery FMD in healthy middle-aged and older men and postmenopausal women. We are the first to measure conduit artery endothelium-independent dilation with curcumin supplementation in humans and determine that these improvements in conduit artery EDD were endothelium specific, as no changes in brachial artery smooth muscle sensitivity to nitric oxide were observed. Additionally, we observed sex-differences in brachial artery FMD responsiveness to curcumin supplementation. Specifically, both men and women had significant improvements but the magnitude of improvement was greater in men compared with postmenopausal women. These latter observations are consistent with recent findings from our laboratory and others that changes in conduit artery endothelial function with interventions may be affected by sex [61–65]. Taken together, these data suggest that curcumin may be a promising therapeutic option to improve age-related vascular endothelial function in middle-aged and older adults.

### Curcumin supplementation and large elastic artery stiffness

Stiffening of the large elastic arteries with age is attributed to a combination of functional and structural wall changes [4, 32, 33]. Arterial functional changes result primarily from increased vascular smooth muscle tone whereas structural wall changes are a consequence of extracellular matrix remodeling, including increased deposition of the load-bearing protein collagen and cross-linking proteins (advanced glycation end products), as well as a reduction and fragmentation of the elasticity conferring protein elastin [4, 32, 34].

A recent preclinical study by our laboratory [51] demonstrated that 4 weeks of curcumin supplementation reversed age-related large elastic artery stiffness (decreased aortic PWV) in mice and that these improvements were associated with reduced collagen and advanced glycation end products in the aorta. In healthy Japanese postmenopausal women, Akazawa et al. [66] reported that 8 weeks of curcumin supplementation improved carotid artery compliance. In contrast, despite improvements in vascular endothelial function, in the present study no changes were observed in regional or local large elastic artery stiffness (aortic PWV or carotid artery compliance, respectively) after 12 weeks of curcumin supplementation in healthy middle-aged and older men and postmenopausal women. Analysis of potential sex differences indicated no improvements in postmenopausal women in the curcumin-supplemented group. Differences in carotid artery compliance outcomes between our study and that of Akazawa et al. [66] may be due to the reduction in carotid systolic blood pressure observed in the latter investigation, explaining the increase in carotid artery compliance but no significant change in β-stiffness, a blood pressure independent index of arterial stiffness [67]. In the present study we did not observe any changes in carotid systolic blood pressure with 12 weeks of curcumin supplementation. Differences in the subject populations studied—middle-aged and older men and women (mostly Caucasian) vs. postmeno-pausal Japanese women—also may have contributed.

Our findings here are consistent with those of several reports suggesting little or no effect of several weeks to months of nutraceutical-based treatment on large elastic artery stiffness in healthy middle-aged and older adults [68–73], including studies concurrently demonstrating improvements in vascular endothelial function in response to the same treatment [74, 75]. Other studies, however, have reported improvements in arterial stiffness with nutraceutical interventions [66, 76, 77]. In some cases, these investigations have shown improve-ments in measures such as carotid artery compliance in the absence of changes in aortic pulse wave velocity (aortic stiffness) [78]. In other instances, aortic stiffness-lowering effects have been reported with longer intervention periods [79]. Lack of improvements in arterial stiffness in studies showing increases in endothelial function may be due to differences in the mechanisms determining those respective vascular functions. Both are influenced by vasodilatory factors (e.g., nitric oxide) [36, 80], but arterial stiffness is determined to a greater extent by the composition of structural proteins in the arterial wall [34, 36, 81]. Structural changes may require a longer treatment period or a different type of intervention (e.g., a stimulus that alters intravascular hemodynamics). Differences in responsiveness between human and animal studies of nutrient-based interventions could be due to a number of factors, including differences in variation of genetic background, metabolism, length of intervention relative to lifespan, and environmental factors.

### Curcumin supplementation and markers of systemic oxidative stress and inflammation

Cell culture and preclinical studies have demonstrated that curcumin has antioxidant and anti-inflammatory properties [38–46]. However, in the present study, no change was observed in circulating markers of oxidative stress (oxidized LDL, TAS, glutathione peroxidase) and inflammation (C-reactive protein, IL-6, TNF-α) with 12 weeks of curcumin supplementation. Although studies in humans evaluating the impact of curcumin supplementation on systemic markers of oxidative stress and inflammation are limited, 4 to 6 weeks of curcumin has been reported to reduce or have no effect on such circulating markers in healthy adults [48, 82]. Moreover, these circulating markers are not consistently altered in intervention studies that improve vascular endothelial function in healthy middle-aged and older adults [74, 78, 83]. The lack of change can be attributed to the relatively low levels of systemic oxidative stress and inflammation that, although typically are greater than levels in healthy young adults, are modest compared with patients with chronic diseases, such as overt CVD [84, 85]. Importantly, recent studies suggest that circulating biomarkers may not be reflective of the local vascular endothelial state in healthy older adults [86, 87]. As such, the observed reduction in vascular oxidative stress, demonstrated by reduced improvement in EDD with vitamin C infusion following curcumin supplementation, provides the most relevant insight regarding the mechanism of action and endothelium-specific antioxidant effects of curcumin supplementation.

### Limitations

To our knowledge there are no acute *in vivo* assessments of pro-inflammatory-mediated suppression of vascular endothelial function as there are for oxidative stress-mediated suppression of endothelial function using vitamin C infusion. The multiple-day administration of salsalate, a nuclear factor κB-inhibiting compound [88], is challenging and not feasible to administer before and after a chronic intervention. Therefore, we are unable to determine if a reduction in pro-inflammatory vascular signaling contributes to improvements in EDD with curcumin supplementation.

Caution should be taken in generalizing the present findings to other supplements containing curcumin or dietary consumption of curcumin, due to the differences in formulations between supplements and the varying absorption/bioavailability of curcumin from other sources. Using the same formulation as our study, Gota et al. [89] demonstrated that circulating curcumin was detectable one hour after consumption of 650 mg Longvida® and peaked at two hours, whereas curcumin was not detectable in the blood following consumption of unformulated curcuminoid extract (>60% curcumin).

Lastly, we studied primarily healthy Caucasian men and women in the present trial. It remains to be determined whether curcumin supplementation improves vascular function in healthy adults of other ethnicities or in individuals with more severe baseline arterial dysfunction due to the presence of major risk factors for CVD or clinically diagnosed CVD.

### Conclusions and perspectives

In healthy middle-aged and older men and postmenopausal women, 12 weeks of curcumin supplementation is well tolerated, and improves resistance and conduit artery endothelial function. Improvement in resistance artery endothelial function is mediated by increases in nitric oxide bioavailability and reductions in vascular oxidative stress. In contrast, curcumin supplementation did not influence large elastic artery stiffness or circulating biomarkers of oxidative stress or inflammation in our sample of late middle-aged and older healthy adults.

Our findings provide evidence for curcumin supplementation as a promising nutraceutical-based treatment for improving nitric oxide-mediated vascular endothelial function and oxidative stress. As such, curcumin is a nutraceutical that may be helpful for maintaining a healthy vascular endothelium with aging, a key process in preventing the development of athero-sclerosis and attendant arterial diseases. For example, in the present study 12 weeks of curcumin supplementation improved conduit artery endothelial function by 1.3% brachial FMD units in healthy middle-aged and older adults, and a 1% improvement in brachial artery FMD units has been associated with a 13% reduction in risk of future cardiovascular outcomes [90]. Similarly, amongst hypertensive individuals, those with higher FBF_ACh_ have a lower incidence of cardiovascular events in the future, suggesting that improved FBF_ACh_ with curcumin may reduce future CVD [91]. However, additional studies are needed to determine the long-term benefits of curcumin in healthy adults, and to examine the efficacy of curcumin supplementation in individuals with CVD or major risk factors for cardiovascular disorders.

## MATERIALS AND METHODS

### Ethics statement

Investigation has been conducted in accordance with the ethical standards and according to the Declaration of Helsinki and national and international guidelines. All procedures were reviewed and approved by the Institutional Review Board at the University of Colorado Boulder. The nature, risks, and benefits of all study procedures were explained to volunteers and their written informed consent was obtained before participation in the study. This study was registered on ClinicalTrials.gov (NCT01968564).

### Participants

Thirty-nine healthy men and postmenopausal women aged 45 to 74 years from Boulder County, Colorado and the surrounding areas were studied. All participants were non-smokers and free of clinical diseases, including peripheral arterial disease (ankle-brachial index >0.90), as determined by medical history, physical examination, blood chemistries, and blood pressure and electrocardiogram at rest and during incremental treadmill exercise. All postmenopausal women were amenorrheic ≥1 year and postmenopausal women ≤56 years of age had a follicular stimulating hormone concentration ≥40 IU/L. Participants demonstrated age-related conduit artery endothelial dysfunction at screening, defined as brachial artery FMD <7%.

### Measurements

All measurements were performed at the University of Colorado Boulder Clinical Translational Research Center (CTRC) after a >12-hour fast (water allowed) from food, caffeine, and dietary supplements, and >24-hour refrainment from alcohol, physical activity, and prescription medications [92]. A single, blinded, investigator performed all primary data acquisition and analysis.

### Participant characteristics

Body mass index and waist and hip circumferences were determined by anthropometry [93]. Percent body fat was measured using dual-energy X-ray absorptionmetry (DEXA; GE Lunar Prodigy Advance). Arterial systolic and diastolic blood pressures were assessed in triplicate over the brachial artery at rest with a semi-automated device (Dinamap XL, Johnson & Johnson). Maximal oxygen consumption (VO_2_ max) was measured during incremental treadmill exercise testing performed to exhaustion (Balke protocol) using open circuit spirometry, as previously described [94]. Physical activity energy expenditure per week was determined by the Community Healthy Activities Model Program for Seniors questionnaire [95].

### Circulating humoral factors

All blood samples were drawn from an intravenous catheter at the cubital vein. The Colorado Clinical and Translational Sciences Institute CTRC Core Laboratory and Boulder Community Hospital Clinical Laboratory performed all blood assays, as previously described [78]. Fasting serum lipids were determined with standard assays. Fasting plasma glucose was measured by reflective spectrophotometry (Ortho Clinical Diagnostics) and fasting plasma insulin and serum adiponectin and leptin by radioimmunoassay (Millipore). HOMA-IR was calculated as [fasting plasma glucose (mg/dL) × fasting plasma insulin (μU/mL)]/405 [96]. Serum follicular stimulating hormone was determined by chemiluminescence (Ortho Clinical Diagnostics). Serum high-sensitivity C-reactive protein was measured by immunoturbidimetry (Beckman Coulter). Serum IL-6 and TNF-α (R&D Systems), and plasma oxidized LDL were assessed by ELISA (Mercodia). Serum TAS and whole blood glutathione peroxidase were measured by oxidative method (Randox Laboratories). Plasma epinephrine and norepinephrine were assessed by high performance liquid chromatography (BioRad) and plasma endothelin-1 (Peninsula Labs) by radioimmunoassay. Serum cortisol was determined by a one-step competitive assay (Beckman Coulter) and serum free fatty acids by enzymatic methods (Wako Chemicals USA).

### Curcumin administration, safety, and tolerability

Participants were randomized to placebo or curcumin supplementation for 12 weeks in a double-blind manner using a blocked randomization scheme stratified for sex (male vs. female). Placebo or curcumin capsules [2000 mg/day Longvida® (∼400 mg curcumin; solid lipid particle formulation)] provided by Verdure Sciences (Noblesville, IN) were taken once every morning while fasted. The Food and Drug Administration categorized curcumin as a supplement for the administration utilized in this study. Every two weeks of the intervention, in-person check-in visits were performed to exchange intervention capsules (precise number of pills were allocated until the participant's next visit) and to assess participant adherence by survey and pill count. Tolerability and side effects were monitored at two-week check-in visits with adverse events documented by the CTRC staff and reported to the Institutional Review Board.

### Dietary analysis

Participants were instructed by the CTRC Boulder registered dietitian to maintain their current caloric intake and avoid foods high in curcumin throughout the intervention. Average daily dietary intake was estimated by three-day diet records at baseline and week 12, and participants repeated the same diet the day prior to all experimental visits. Diet records were analyzed by the CTRC Boulder registered dietitian using Nutrition Data System for Research.

### Resistance artery endothelial function

Resistance artery EDD and endothelium-independent dilation were determined as FBF_ACh_ (1, 2, 4, and 8 μg/100 mL forearm volume/min for 3.5-4 minutes per dose; Bausch and Lomb) and FBF_SNP_ (0.5, 1, and 2 μg/100 mL forearm volume/min for 3.5-4 minutes per dose; Marathon Pharmaceuticals LLC), respectively, using strain gauge venous occlusion plethysmography (A16 Arterial Inflow System, D.E. Hokanson) as previously described [6, 97]. To assess resistance artery nitric oxide-mediated EDD, FBF_ACh_ in the absence and presence of the nitric oxide synthase inhibitor, L-NMMA (10 minute loading dose of 5 mg/minute at 2.5 mL/minute and maintenance dose of 1 mg/minute at 0.5 mL/minute; Bachem), was measured. Resistance artery oxidative stress-mediated suppression of EDD was determined by FBF_ACh_ in the absence and presence of the antioxidant vitamin C (10 minute loading dose of 25 mg/minute at 2.5 mL/minute and maintenance dose of 25 mg/minute at 0.5 mL/minute; Mylan Institutional LLC). FBF values are reported as individual dose responses and AUC.

### Conduit artery endothelial function

Conduit artery EDD and endothelium-independent dilation were determined by brachial artery FMD (using a five-minute forearm cuff) and brachial artery diameter change following 0.4 mg sublingual nitroglycerin, respectively, using high-resolution ultrasonography (Toshiba Xario XG) as previously described [92, 98, 99]. Brachial artery FMD was measured at baseline, week 4, and week 12, and brachial artery dilation to nitroglycerin at baseline and week 12. Brachial artery FMD and dilation to nitroglycerin are reported as percentage and absolute (mm) change from baseline diameter [92]. Brachial artery FMD shear rate was calculated as [8 × mean velocity (m/s)]/occlusion diameter (m) [92]. Brachial artery diameters and blood velocities were captured and analyzed by Vascular Research Tools 5.10.9 (Medical Imaging Applications).

### Large elastic artery stiffness

Large elastic artery stiffness was assessed regionally via aortic PWV and locally via carotid artery compliance as previously described [100]. Briefly, central (aortic: carotid to femoral) and peripheral (carotid to radial) PWV was determined by applanation tonometry with simultaneous electrocardiogram gating of the R-wave to measure the time delay between the foot of the carotid and femoral or radial arterial pressure waves and augmentation index was measured by applanation tonometry of the common carotid or radial artery (Non-Invasive Hemodynamics Workstation, Cardiovascular Engineering Inc.). PWV and augmentation index were calculated by the Non-Invasive Hemodynamics Workstation as the distance between arterial sites (cm) divided by the arterial pressure wave transit time at each site (s), and the Δpressure (mmHg) divided by the pulse pressure (mmHg) × 100, respectively [100].

Carotid artery compliance was assessed using ultrasonography (Toshiba Xario XG) to measure arterial diameter from end-systole to end-diastole while simultaneously measuring carotid arterial pressure changes via applanation tonometry as previously described [24]. Carotid artery compliance was calculated as {3.141592 × 2 × carotid diastolic diameter (mm) × [carotid systolic − diastolic diameter (mm)] + [carotid systolic − diastolic diameter (mm)]^2^}/[4 × carotid pulse pressure (mmHg)] [101] and β-stiffness as Ln[carotid systolic blood pressure (mmHg)/carotid diastolic blood pressure (mmHg)]/{[carotid systolic diameter − carotid diastolic diameter (mm)]/carotid diastolic diameter (mm)} [67]. Carotid artery diameters were captured and analyzed by Vascular Research Tools 5.10.9 (Medical Imaging Applications).

### Data analysis

Statistical analysis was performed with IBM SPSS 23 and G*Power 3.1. Data normality was assessed with the Shapiro-Wilk test and non-normal variables were log base 10 transformed for statistical analysis. Outliers (≥3 standard deviations) were replaced with the group mean. An independent t-test was performed to assess group differences at baseline. A mixed-model ANOVA was performed to identify group (curcumin vs. placebo) by time [week 0, (4), and 12] interactions for all primary outcomes and clinical characteristics. To determine if there were sex-differences in the curcumin group after 12 weeks of supplementation, a mixed-model ANOVA was performed to identify any sex (men vs. women) by time interactions for all primary outcomes. In the case of a significant effect of curcumin supplementation on FBF_ACh_, a mixed-model ANOVA was performed to identify group by time interactions for nitric oxide (via L-NMMA) and oxidative stress (via vitamin C)-mediated EDD. In the case of significant interactions, a paired t-test was performed for within-group comparisons with Bonferroni correction. The acute effects on FBF_ACh_ with co-infusion of L-NMMA and vitamin C were assessed by paired t-tests with Bonferroni correction. Sample size was estimated based on our laboratory's previous lifestyle intervention studies, using the primary outcome with the lowest effect size (FBF_ACh_: 0.7) to detect significant group differences [83, 102–104]. Data are expressed as mean±standard error (SE). Statistical significance was set at α<0.05.

## SUPPLEMENTARY MATERIAL TABLES


