# Supplementary material for: Curcumin supplementation improves vascular endothelial function in healthy middle-aged and older adults by increasing nitric oxide bioavailability and reducing oxidative stress
Source: Aging (Albany NY). 2017 Jan 3;9(1):187–205. doi: 10.18632/aging.101149 (PMC5310664; doi:10.18632/aging.101149)
Supplement: Supplementary file 1 [file aging-09-0187-s001.pdf]

SUPPLEMENTARY MATERIAL

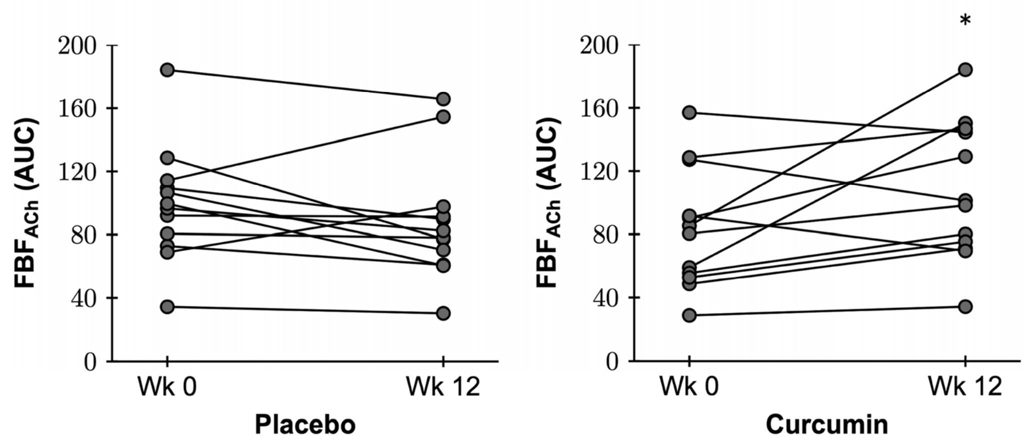

Supplemental Figure 1. Forearm blood flow in response to acetylcholine (FBF_{ACh}) area under the dose-response curve (AUC) for individuals at week 0 and after 12 weeks of placebo or curcumin supplementation. Group by time P=0.02, *P=0.03 vs. curcumin week 0.

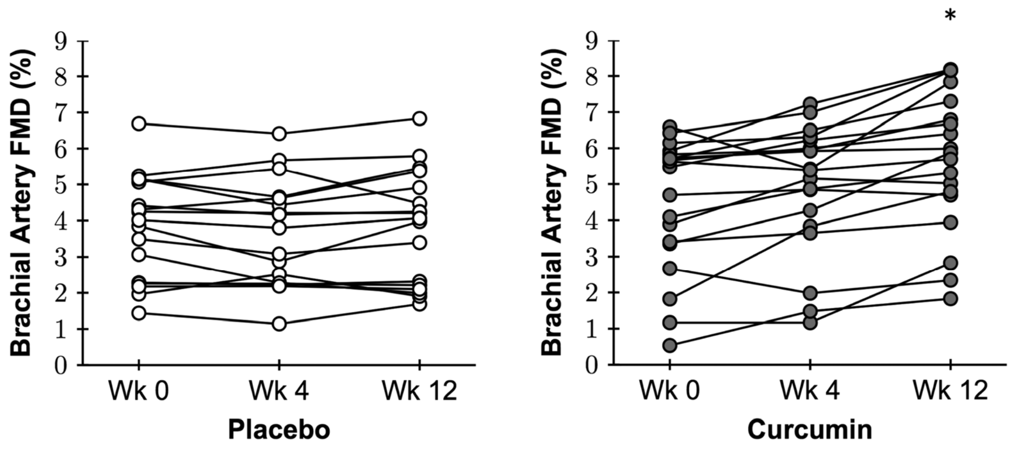

Supplemental Figure 2. Brachial artery flow-mediated dilation (FMD) expressed as percent change for individuals at week 0 and after 4 and 12 weeks of placebo or curcumin supplementation. Group by time P=0.001, *P=0.001 vs. curcumin week 0.

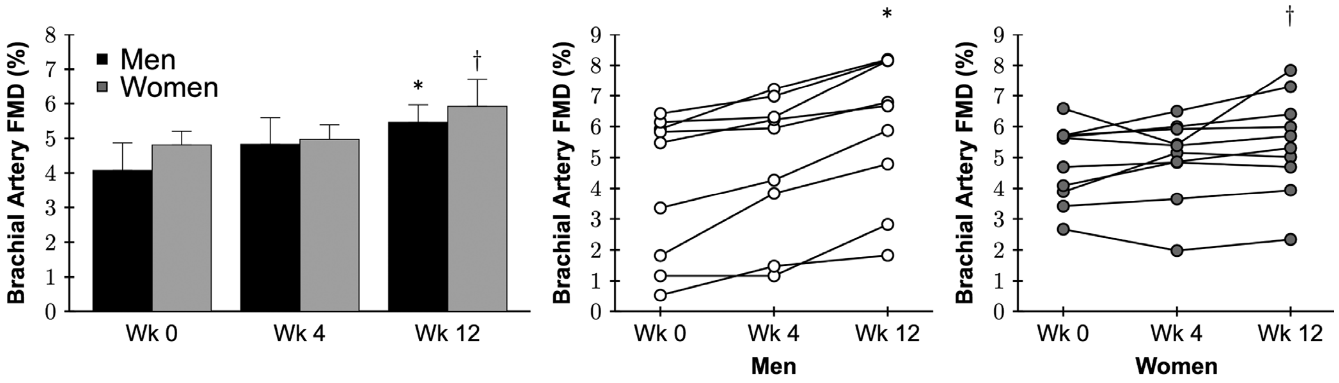

Supplemental Figure 3. Brachial artery flow-mediated dilation (FMD) expressed as percent change for men and women (*left*) and individual responses separated by sex (*middle and right*) at week 0 and after 4 and 12 weeks of curcumin supplementation. Data are mean±SE; Sex by time P=0.001, *P=0.001 vs. men week 0, †P=0.01 vs. women week 0.

Supplemental Table 1. Circulating humoral factors in subset of participants.

	Placebo		Curcumin	
	Week 0	Week 12	Week 0	Week 12
Interleukin-6, pg/mL[L]	0.84±0.19	1.64±0.48	0.90±0.12	1.16±0.20
TNF-α, pg/mL[L]	1.13±0.17	1.11±0.17	0.85±0.07	0.97±0.07
Oxidized LDL, U/L	30±2	33±2	33±3	33±3
Total antioxidant status, mmol/L	1.48±0.05	1.47±0.06	1.44±0.05	1.45±0.04
Glutathione peroxidase, U/L	7497±555	7397±639	7591±497	7346±556
Epinephrine, pg/mL	31.72±5.47	28.36±4.05	24.85±2.66	25.85±3.79
Norepinephrine, pg/mL	286±23	315±40	303±30	323±48
Endothelin-1, pg/mL	5.29±0.29	4.83±0.17	5.90±0.47	5.42±0.33
Cortisol, µg/mL	9.42±0.73	9.33±0.81	8.29±0.69	8.86±0.95
Free fatty acids, µmol/L	433±27*	415±66	546±39	486±31
Adiponectin, µg/mL	10.8±1.8	10.7±2.1	9.7±1.3	10.0±1.4
Leptin, ng/mL[L]	6.1±2.0	6.8±2.1	8.7±1.9	11.2±2.8
Insulin, µU/mL[L]	8±1	8±1	7±1	8±1
HOMA-IR, U[L]	1.6±0.2	1.6±0.2	1.5±0.2	1.6±0.2

Data are mean ± SE; TNF, tumor necrosis factor; LDL, low-density lipoprotein; HOMA-IR, homeostasis model assessment of insulin resistance;

[L]Data log transformed for statistical analysis; *P=0.03 vs. curcumin week 0

Supplemental Table 2. Dietary Intake.

	Placebo		Curcumin	
	Week 0	Week 12	Week 0	Week 12
Total daily energy (kcal)	1962±128	1945±83	2092±206	1974±198
Daily relative carbohydrate (% of total kcal)	41±2	42±2	44±2	44±2
Daily relative protein (% of total kcal)	20±1*	19±1	16±1	18±1
Daily relative fat (% of total kcal)	35±2	35±2	37±2	36±2

Data are mean±SE; *P=0.02 vs. curcumin week 0
